# Perceived discrimination and psychosocial adaptation among poverty-relocated children with subjective socioeconomic status and core self-evaluations as mediating and moderating factors

**DOI:** 10.3389/fpsyg.2026.1767109

**Published:** 2026-06-04

**Authors:** Xiang Zhang, Zhuotao Fang

**Affiliations:** School of Educational Sciences, Minzu Normal University of Xingyi, Xingyi, Guizhou, China

**Keywords:** children relocated to alleviate poverty, core self-evaluations, perceived discrimination, psychosocial adaptation, subjective socioeconomic status

## Abstract

**Introduction:**

A large number of children have been relocated through poverty-alleviation programs into newly established resettlement communities in China. These children have experienced substantial environmental changes and may face significant challenges in psychosocial adaptation. The present study examined the mediating role of subjective socioeconomic status and the moderating role of core self-evaluations in the association between perceived discrimination and psychosocial adaptation among children relocated through poverty-alleviation initiatives.

**Methods:**

A cross-sectional survey was conducted among 1,426 Chinese children from six schools in Guizhou Province. Perceived discrimination was treated as the independent variable, subjective socioeconomic status as the mediating variable, core self-evaluations as the moderating variable, and psychosocial adaptation as the dependent variable. Mediation and moderation analyses were conducted using Hayes’s PROCESS macro in SPSS.

**Results:**

The results indicated that perceived discrimination was negatively associated with psychosocial adaptation and with both subjective socioeconomic status and core self-evaluations. Subjective socioeconomic status was positively associated with psychosocial adaptation and was linked to the association between perceived discrimination and psychosocial adaptation. In addition, core self-evaluations moderated the association between perceived discrimination and psychosocial adaptation. The observed interaction pattern may be interpreted cautiously as being consistent with a protective-reactive form of moderation.

**Discussion:**

These findings provide insights into the psychological processes and individual differences associated with the psychosocial adaptation of children relocated through poverty-alleviation programs and may inform interventions aimed at supporting their adjustment in resettlement contexts.

## 1 Introduction

Poverty-alleviation relocation represents one of the largest state-led social mobility programs implemented in contemporary China (Wang et al., 2024). As a core component of the national targeted poverty-reduction strategy, the policy aims to relocate populations living in environmentally fragile or economically unsustainable regions to newly constructed resettlement communities where infrastructure, employment opportunities, and public services are more accessible (Cheng, 2023a). During the Thirteenth Five-Year Plan period (2016–2020), approximately 9.6 million residents were relocated through this program (Wang et al., 2024; Zhao et al., 2026). A substantial proportion of the relocated population consists of school-aged children who moved together with their families from rural mountainous areas to resettlement communities located near towns or cities.

For children, relocation represents a major ecological transition that extends far beyond physical movement. Their family routines, schooling environments, peer networks, and community contexts change simultaneously. Such transitions often require children to renegotiate social relationships, adapt to new institutional norms, and reconstruct their sense of belonging within unfamiliar environments (Zhao et al., 2026). These adjustments can place considerable psychological demands on young people and make psychosocial adaptation a central developmental challenge for children who experience poverty-alleviation relocation.

Psychosocial adaptation refers to the process through which individuals adjust their psychological functioning and behavioral patterns in response to environmental change, allowing them to achieve a renewed equilibrium within a social context (Londono and McMillan, 2015). Among children relocated through poverty-alleviation programs, this adaptive process typically involves multiple domains, including interpersonal adaptation, environmental adaptation, daily life adaptation, academic adaptation, and psychological adjustment (Zhang et al., 2007; Zhang, 2022). In the present study, psychosocial adaptation is operationalized as the overall level of adjustment across these five domains, measured using a validated multidimensional scale.

Research on migration psychology provides an important starting point for understanding the present population, but poverty-alleviation relocation should not be conflated with generic migrant movement (Ababiel et al., 2025; Zhu et al., 2023). In China, most research on children’s migration has focused on liudong ertong in the context of labor-driven and rural-to-urban migration (Cao, 2024). This literature typically conceptualizes adaptation in terms of acculturation, school adjustment, and the negotiation of urban belonging under the constraints of the hukou system (Cao, 2024). Rural-to-urban migrant children are commonly discussed in relation to accommodation to urban culture, maintenance of ties to rural culture, and the effects of outsider status and urban–rural discrimination on adjustment (Liu, 2025). Poverty-alleviation relocation overlaps with this literature but is not identical to it. Unlike labor-driven rural-to-urban migration initiated primarily by families seeking employment opportunities, poverty-alleviation relocation is a state-led resettlement policy through which families move from remote and resource-constrained areas to designated communities near towns or cities (Wang and Mesman, 2015). For children, this form of mobility is typically low in personal agency and embedded in policy implementation rather than individual choice. Recent studies on poverty-alleviation relocation therefore emphasize abrupt changes in living space, livelihoods, community structure, and social integration, including psychological identity and post-resettlement support, rather than acculturation alone (Garriga et al., 2023; Wang and Mesman, 2015). Accordingly, poverty-relocated children may share with rural-to-urban migrant children the challenge of adapting to a new urbanizing environment, but they may also face distinctive post-relocation social integration pressures because poverty-alleviation relocation is a state-led resettlement process in which migrants must renegotiate belonging, community attachment, and social position in newly formed resettlement settings (Cheng, 2023a; Yang et al., 2025). Their adaptation should therefore be understood not simply as urban acculturation, but as psychosocial adjustment within a policy-driven, relatively low-agency resettlement context in which belonging, recognition, and perceived social standing must be renegotiated (Cheng, 2023a).

Within this context, perceived discrimination is conceptualized in the present study as status-based discrimination in the resettlement environment. For children relocated through poverty-alleviation programs, unfair treatment is unlikely to stem primarily from ethnicity or nationality, as is often the case in international migration research (Yu et al., 2022). Rather, it is more likely to reflect their socially marked lower-status position in the receiving environment (Cheng, 2023a). In schools and resettlement communities, children from relocated families may be viewed through overlapping cues associated with poverty, rural origin, and resettlement background (Li and Jiang, 2018). In the present study, these are not treated as separate discrimination constructs, but as interrelated social markers through which children may experience devaluation, exclusion, or unfair treatment (Liu et al., 2014). Accordingly, the focal construct examined here is children’s overall perception of being treated as lower in status in the resettlement context, rather than any single subtype of stigma considered in isolation.

To explain how perceived discrimination becomes associated with psychosocial adaptation in the resettlement context, the present study draws on the context of reception framework, which emphasizes that adaptation in a new environment is shaped by how individuals are socially evaluated and positioned by others in the receiving context (Bankston and Zhou, 2021). In the present study, this framework is specified in two propositions. First, social evaluation signals in the receiving context, such as perceived discrimination, shape individuals’ perceived social positioning. In this sense, discrimination is not only an interpersonal experience but also a status-relevant signal that informs how children locate themselves within the local social hierarchy. Second, the impact of these social evaluation signals depends on individuals’ evaluative resources. That is, the extent to which discrimination is linked to adaptation varies depending on how individuals interpret and respond to these experiences. Corresponding to these propositions, subjective socioeconomic status (SSS) is conceptualized as a social-positioning mechanism through which perceived discrimination becomes linked to psychosocial adaptation, whereas core self-evaluations are conceptualized as a personal evaluative resource that conditions this association (Judge et al., 2003).

From this perspective, SSS represents an internalized appraisal of one’s relative social standing in the new environment. Importantly, subjective socioeconomic status is not treated here as a substitute for objective household socioeconomic conditions. Rather, it is conceptualized as children’s perceived relative standing in the social environment, which may be shaped by, but is not reducible to, objective socioeconomic resources (Quon and McGrath, 2014). The present study therefore focuses on the potential relevance of perceived social rank in the association between perceived discrimination and psychosocial adaptation, while recognizing that objective family background and relocation-related characteristics may also be associated with this process. Research on symbolic interactionism suggests that children develop self-understandings partly through reflected appraisals and everyday social feedback (Brown and Lohr, 1987). When children repeatedly encounter exclusion, disrespect, or subtle devaluation, these experiences may communicate more than momentary rejection; they may also imply that the child occupies a lower-valued position in the surrounding social world (Cao, 2024). In a resettlement context, such signals may be especially salient because relocation often places children into new comparison environments where differences in family background, lifestyle, and perceived status become more visible (Cao, 2024). Under these conditions, perceived discrimination may be associated with lower subjective socioeconomic status, which may in turn be linked to poorer psychosocial adaptation. On this basis, the present study proposes the following hypothesis:

*H_1_*: Subjective socioeconomic status is linked to the association between perceived discrimination and psychosocial adaptation among children relocated through poverty-alleviation programs.

At the same time, children may differ in how strongly they are affected by the same discriminatory experiences. In the present framework, core self-evaluations (CSE) are conceptualized as a personal evaluative resource that conditions children’s responses to social-positioning threats (Judge et al., 2003). CSE reflects individuals’ fundamental appraisal of their own worth, competence, and capacity to influence life outcomes (Chang et al., 2012; Elliott et al., 2012). Children with higher CSE generally possess stronger confidence and more positive self-views, which may support better psychosocial functioning under relatively favorable or moderately stressful conditions (Elliott et al., 2012). However, these same positive self-appraisals may also heighten expectations of fairness, recognition, and social acceptance. When such expectations are contradicted by discriminatory experiences, the discrepancy between self-appraisal and social feedback may become especially salient, thereby altering the strength of the association between perceived discrimination and psychosocial adaptation (Gu et al., 2024; Swann et al., 2007).

Because prior evidence on this specific interaction among poverty-relocated children remains limited, the present study focuses on the existence of moderation as the primary hypothesis, while treating the exact form of the interaction more cautiously. Accordingly, the present study conceptualizes psychosocial adaptation under poverty-alleviation relocation as the joint product of children’s social positioning in the receiving context and their personal evaluative resources. Perceived discrimination is expected to be associated with poorer adaptation partly because it is linked to lower subjective socioeconomic status (Schmitt et al., 2014), while the strength of this association may vary depending on children’s core self-evaluations (Quon and McGrath, 2014). In this way, the mediation and moderation components are treated not as separate theoretical strands, but as complementary mechanisms within a single integrated framework.

*H_2_*: Core self-evaluations moderate the association between perceived discrimination and psychosocial adaptation among children relocated through poverty-alleviation programs.

**Figure 1:**
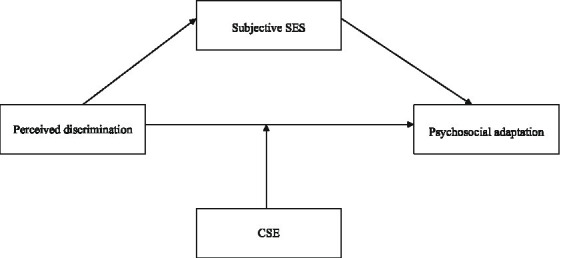
Mediation and moderation model. SES = Socioeconomic status; CSE = Core self-evaluations.

Figure 1 presents the hypothesized model. Guided by the context of reception framework, the present study examines whether subjective socioeconomic status links perceived discrimination to psychosocial adaptation and whether core self-evaluations condition the strength of this association among children relocated through poverty-alleviation programs.

## 2 Methods

### 2.1 Participants

The study was conducted in three prefecture-level regions of Guizhou Province, China, where large-scale poverty-alleviation relocation programs have been implemented. Six public schools serving resettlement communities were selected as research sites, including three primary schools and three junior secondary schools. These schools primarily enroll students whose families were relocated through the national poverty-alleviation relocation program.

In the present study, “relocated children” refers to students whose families had been officially relocated from rural poverty-stricken areas to newly established resettlement communities near towns or urban peripheries as part of the government’s targeted poverty-alleviation relocation policy. Most relocated families had moved from remote mountainous villages to newly constructed residential communities with improved housing, infrastructure, and access to public services. All participating students had lived in the resettlement communities for at least one academic semester prior to the survey.

A cluster sampling strategy was used to recruit participants. Students from Grades 4–6 in primary schools and Grades 7–9 in junior secondary schools were invited to participate. In total, 1,500 relocated children were surveyed. Data collection took place between 1 April and 30 April 2022. Because all participants were minors (aged 9–17 years; *M* = 12.13, SD = 1.75), written electronic informed consent was first obtained from parents or legal guardians through the schools’ secure online platform. After parental consent was obtained, students provided electronic assent by selecting an “I agree” option after reading a brief description of the study’s purpose, voluntary nature, confidentiality safeguards, and right to withdraw at any time. Consent records were automatically time-stamped and stored on an encrypted server, and no personally identifiable information was collected.

A total of 1,426 valid questionnaires were obtained, yielding a response rate of 95.06%. The final sample consisted of 621 boys (43.5%) and 805 girls (56.5%). Among the participants, 758 students (53.16%) were enrolled in primary schools and 668 students (46.84%) were enrolled in junior secondary schools. Because participants were recruited from six schools using cluster sampling, observations may not be entirely independent. This sampling structure was therefore considered in the statistical analysis when interpreting the results.

### 2.2 Measurements

#### 2.2.1 Psychosocial adaptation of children relocated to alleviate poverty

Psychosocial adaptation was measured using a 24-item scale developed by Zhang (2022). All items were rated on a five-point Likert scale ranging from 1 (strongly disagree) to 5 (strongly agree). Following the reverse scoring of relevant items, mean scores were calculated for each subdimension as well as for the overall scale, with higher scores indicating more favorable levels of psychosocial adaptation. In the present study, the internal consistency of the scale was high, with a Cronbach’s *α* of 0.88.

#### 2.2.2 Perceived discrimination

Perceived discrimination was assessed using a six-item questionnaire revised by Shen et al. (2009). Each item was rated on a five-point Likert scale, with responses ranging from 1 (strongly disagree) to 5 (strongly agree). The mean score across all items was calculated to represent the level of perceived discrimination, with higher scores indicating stronger perceptions of being treated unfairly or devalued. In the present study, this scale was used as a global indicator of children’s perceived status-based unfair treatment in the resettlement context. That is, the measure was intended to capture children’s overall perception of devaluation and exclusion associated with their socially marked position after relocation, rather than to distinguish among separate forms of poverty stigma, rural–urban status discrimination, and resettlement identity stigma (Li and Jiang, 2018). In the present study, these sources were treated as overlapping status-related cues within a broader construct of perceived discrimination. The scale demonstrated good internal consistency, with a Cronbach’s *α* of 0.81.

#### 2.2.3 Core self-evaluations

Core self-evaluations were measured using the 10-item Chinese version of the Core Self-Evaluations Scale, adapted by Du et al. (2012) based on the original scale developed by Judge et al. (2003). Each item was rated on a five-point Likert scale, with higher average scores indicating higher levels of core self-evaluations. Negatively worded items were reverse-coded to ensure that higher scores consistently reflected more positive self-evaluations. In the present study, the scale demonstrated acceptable internal consistency, with a Cronbach’s α of 0.76.

#### 2.2.4 Subjective socioeconomic status

Subjective socioeconomic status was measured using the MacArthur Scale of Subjective Social Status (Adler and Stewart, 2007). Participants were shown an image of a ladder with ten rungs representing different positions in the social hierarchy, where higher rungs indicate higher perceived socioeconomic standing. Children were asked to place an “X” on the rung that best represented their family’s overall social and economic standing relative to others in society, considering their living conditions and family background. Higher ladder positions indicate higher perceived socioeconomic status. The MacArthur ladder is widely used as a single-item measure capturing individuals’ perceived relative social status and has been shown to be associated with a range of psychological and health outcomes.

### 2.3 Statistical analysis

All statistical analyses were conducted using SPSS 22.0 and Mplus 8.0. The analytical procedure consisted of several steps. First, the potential influence of common method variance was examined using Harman’s single-factor test. Second, descriptive statistics and Pearson correlation analyses were conducted to examine the associations among psychosocial adaptation, perceived discrimination, subjective socioeconomic status, and core self-evaluations. Third, confirmatory factor analyses were conducted to evaluate the measurement validity of the study constructs and to compare competing measurement models. Finally, the hypothesized mediation and moderation effects were tested using Hayes’s PROCESS macro (version 2.13.2). Specifically, PROCESS Model 5 was applied to examine whether subjective socioeconomic status mediated the association between perceived discrimination and psychosocial adaptation, while core self-evaluations moderated the direct relationship between perceived discrimination and psychosocial adaptation. Gender, age, and school level were included as control variables in the analyses because prior research has shown that children’s school and psychological adaptation may vary by gender, developmental stage, and grade/school level (Chen et al., 2024; Goodman et al., 1993). At the same time, we acknowledge that other plausible background factors, such as household economic conditions, parental education, time since relocation, and prior residence characteristics, may also be related to both subjective socioeconomic status and psychosocial adaptation. Because these variables were not available in the present dataset, the analyses should not be interpreted as isolating the unique association of subjective socioeconomic status independent of all objective socioeconomic and relocation-history factors. To evaluate the statistical significance of indirect and interaction effects, bias-corrected bootstrap confidence intervals were computed based on 5,000 resamples. A 95 percent confidence interval was adopted to assess the robustness of the observed effects.

## 3 Results

### 3.1 Common method bias testing

Because all variables were collected using self-report questionnaires from the same participants, common method variance (CMV) may pose a potential concern. To reduce this risk, several procedural remedies were implemented during data collection. Standardized administration procedures were applied across all survey sites. Trained investigators provided identical instructions, questionnaires were completed within a single session, and responses were collected immediately after completion to minimize discussion or external influence.

To statistically examine the potential influence of CMV, Harman’s single-factor test was conducted following the procedure described by Podsakoff et al. (2003). An exploratory factor analysis revealed eight factors with eigenvalues greater than one. The first unrotated factor accounted for 21.84% of the total variance, which is below the commonly referenced threshold of 40% (Podsakoff et al., 2003). These results suggest that common method variance is unlikely to substantially bias the results. However, because Harman’s single-factor test has been criticized as a relatively insensitive diagnostic method, the presence of common method bias cannot be entirely ruled out (Kock et al., 2021).

### 3.2 Descriptive statistics and correlation coefficients of variables

Descriptive statistics and Pearson correlation coefficients for psychosocial adaptation, perceived discrimination, subjective socioeconomic status, and core self-evaluations are presented in Table 1. The results revealed that psychosocial adaptation was significantly and negatively correlated with perceived discrimination (*p* < 0.01). In addition, perceived discrimination exhibited significant negative correlations with both subjective socioeconomic status and core self-evaluations (*p* < 0.01). Conversely, psychosocial adaptation was positively associated with both subjective socioeconomic status and core self-evaluations (*p* < 0.01). Distribution diagnostics indicated that skewness and kurtosis values for all variables fell within acceptable ranges, suggesting that the assumption of normality was not seriously violated.

**Table 1:** Results of descriptive statistics and correlation analysis of variables.

Variable	1	2	3	4	M	SD	Skewness	Kurtosis
1. Psychosocial adaptation	1				4.34	0.49	−0.72	0.64
2. Perceived discrimination	−0.46**	1			2.14	0.84	0.81	0.37
3. Subjective SES	0.15**	−0.16**	1		4.63	1.48	−0.48	−0.32
4. CSE	0.43**	−0.49**	0.15**	1	3.44	0.7	−0.36	−0.41

*N* = 1,426, SES = Socioeconomic Status; CSE = Core Self-evaluations, ^**^p < 0.01.

### 3.3 Measurement model

To examine the discriminant validity among the study variables, a series of confirmatory factor analyses (CFA) were conducted to compare several competing measurement models. Four models were estimated. The one-factor model assumed that all measurement items loaded onto a single latent factor, representing the most constrained model. The two-factor model combined perceived discrimination and psychosocial adaptation into one factor while retaining core self-evaluations as a separate factor. The three-factor model specified perceived discrimination, psychosocial adaptation, and core self-evaluations as three distinct latent constructs. Finally, the four-factor model represented the hypothesized measurement structure in which each construct was modeled as a separate latent factor.

The results are presented in Table 2. The one-factor model showed poor fit to the data (χ^2^ = 5124.36, df = 594, CFI = 0.58, TLI = 0.54, RMSEA = 0.092), suggesting that common method variance was unlikely to account for the relationships among the variables. Model fit improved progressively as additional factors were introduced. The hypothesized four-factor model demonstrated the best fit to the data (χ^2^ = 1842.15, df = 588, CFI = 0.91, TLI = 0.90, RMSEA = 0.046, SRMR = 0.041), outperforming all alternative models. These results support the discriminant validity of the study constructs.

**Table 2:** Comparison of competing measurement models.

Model	χ^2^	df	χ^2^/df	CFI	TLI	RMSEA	SRMR
One-factor model	5124.36	594	8.63	0.58	0.54	0.092	0.084
Two-factor model	3687.21	593	6.22	0.71	0.68	0.074	0.071
Three-factor model	2458.73	591	4.16	0.86	0.84	0.054	0.056
Four-factor model (theoretical)	1842.15	588	3.13	0.91	0.9	0.046	0.041

One-factor model: all measurement items loaded onto a single latent factor. Two-factor model: perceived discrimination and psychosocial adaptation were combined into one factor, with core self-evaluations specified as a separate factor. Three-factor model: perceived discrimination, psychosocial adaptation, and core self-evaluations were specified as three distinct latent constructs. Four-factor model (theoretical): perceived discrimination, psychosocial adaptation, core self-evaluations, and subjective socioeconomic status were specified as four separate constructs.

### 3.4 Mediation and moderation analyses

After standardizing all variables, Model 5 of Hayes’s PROCESS macro was employed to examine the hypothesized mediation and moderation patterns. Gender (dummy coded) and age were included as control variables. In the model specification, psychosocial adaptation was designated as the dependent variable, perceived discrimination as the independent variable, subjective socioeconomic status as the mediating variable, and core self-evaluations as the moderating variable.

**Figure 2:**
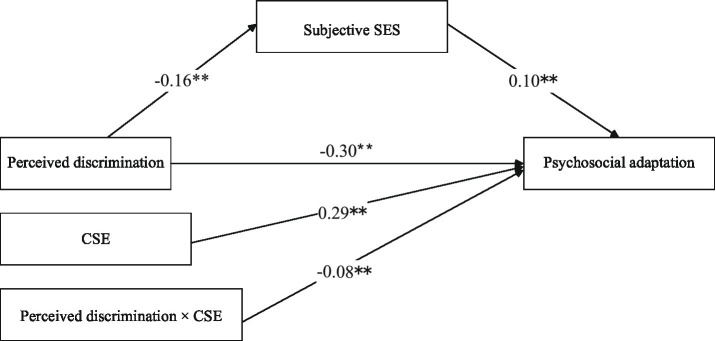
Path diagram of the indirect association through subjective SES and the moderation by CSE. SES = Socioeconomic status; CSE = Core self-evaluations, *p* < 0.01.

As presented in Table 3 and illustrated in Figure 2, perceived discrimination was significantly associated with subjective socioeconomic status (*β* = −0.16, *p* < 0.01), and subjective socioeconomic status was significantly associated with psychosocial adaptation (*β* = 0.10, *p* < 0.01). Moreover, perceived discrimination was significantly and negatively associated with psychosocial adaptation (*β* = −0.30, *p* < 0.01). In addition, the interaction term between perceived discrimination and core self-evaluations was significantly associated with psychosocial adaptation (*β* = −0.08, *p* < 0.01), indicating a statistically significant moderation pattern. These results were consistent with the proposed mediation and moderation hypotheses.

**Table 3:** Analysis of mediating and moderating effects.

**Outcome variable**	**Predictor variable**	**Model 1 *β* (t)**	**Model 2 β (t)**
Subjective SES (Mediator)	Perceived discrimination	−0.16** (−6.02)	—
	*R* ^2^	0.040	—
Psychosocial adaptation (Outcome)	Subjective SES	0.10** (2.95)	0.10** (2.94)
	Perceived discrimination	−0.30** (−11.62)	−0.29** (−11.20)
	CSE	0.29** (6.96)	0.28** (6.80)
	Perceived discrimination × CSE	—	−0.08** (−2.84)
	*R* ^2^	0.284	0.290
	Δ*R*^2^	—	0.006**

SES = Socioeconomic Status; CSE = Core Self-evaluations, Standardized coefficients (β) are reported, Model 1 includes main effects only; Model 2 includes the interaction term, ΔR^2^ indicates the increase in explained variance after adding the interaction term, ^**^p < 0.01.

To assess the statistical significance of the indirect association and the moderation pattern, a bias-corrected bootstrap procedure with 5,000 resamples was employed to generate 95% confidence intervals. An association was considered statistically significant when the confidence interval did not include zero. The indirect association between perceived discrimination and psychosocial adaptation through subjective socioeconomic status was −0.02, with a 95% confidence interval of [−0.031, −0.009], indicating a statistically significant indirect association. The indirect effect accounted for 6.25% of the total effect, indicating a relatively small but non-negligible pathway.

In addition, the confidence interval for the interaction term between perceived discrimination and core self-evaluations did not include zero, suggesting that core self-evaluations significantly moderated the association between perceived discrimination and psychosocial adaptation. As shown in Table 3, the interaction term contributed an additional ΔR^2^ = 0.006 (*p* < 0.01), indicating a small but statistically significant increase in explained variance. Taken together, these results were consistent with the hypothesized model in which subjective socioeconomic status was linked to the association between perceived discrimination and psychosocial adaptation, while core self-evaluations moderated the direct association between perceived discrimination and psychosocial adaptation. Accordingly, both H1 and H2 were supported.

**Figure 3:**
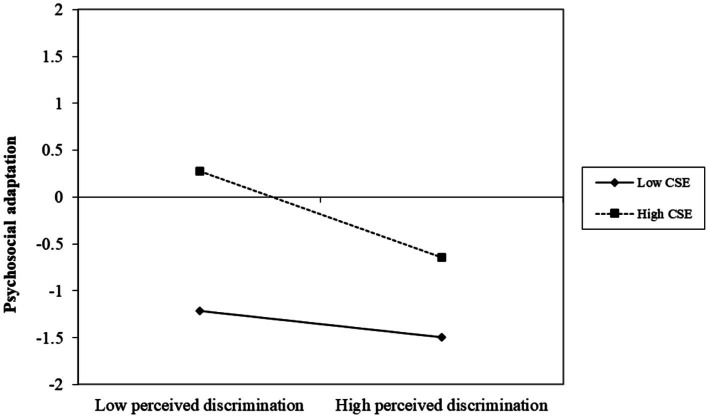
Moderation of the relationship between perceived discrimination and psychosocial adaptation by CSE. CSE = Core self-evaluations.

To further clarify the moderating role of core self-evaluations, a simple slope analysis was conducted by categorizing participants into high and low core self-evaluation groups, defined as one standard deviation above and below the mean, respectively. As illustrated in Figure 3, when core self-evaluations were low (M-1SD), increases in perceived discrimination were significantly associated with a decline in psychosocial adaptation (*β*_simple_ = −0.22, *t* = −6.96, *p* < 0.01). When core self-evaluations were high (M + 1SD), this negative association became even more pronounced (*β*_simple_ = −0.38, *t* = −10.16, *p* < 0.01). These results suggest that perceived discrimination showed a stronger negative association with psychosocial adaptation among children with higher levels of core self-evaluations, whereas this effect is less pronounced among those with lower core self-evaluations. In other words, as the level of perceived discrimination increases, the buffering function of core self-evaluations weakens. The protective role of core self-evaluations is most evident when perceived discrimination is relatively low. This observed interaction pattern can be interpreted as an individual-level moderation process in which positive self-appraisals appear to become less protective when discriminatory experiences strongly contradict children’s expectations of fairness and recognition. Given the limited prior evidence on the exact shape of this interaction in poverty-relocated children, this interpretation should be understood as a theoretically informed account of the obtained pattern rather than as a uniquely pre-specified prediction.

## 4 Discussion

Interpreted through the context of reception framework, the present findings provide support for the two propositions specified in the Introduction. First, the finding that perceived discrimination was associated with psychosocial adaptation both directly and indirectly through subjective socioeconomic status is consistent with the proposition that social evaluation signals in the receiving context shape individuals’ perceived social positioning. In this sense, discriminatory experiences appear to become consequential in part because they inform how children locate themselves within the social hierarchy of the resettlement environment. Second, the moderating role of core self-evaluations supports the proposition that the impact of these social evaluation signals depends on individuals’ evaluative resources. The strength of the association between perceived discrimination and psychosocial adaptation varied across levels of core self-evaluations, suggesting that children differ in how strongly they are affected by status-relevant social feedback depending on how they interpret and respond to such experiences. Because prior evidence did not provide a sufficiently strong basis for specifying the exact form of this interaction *a priori* in poverty-relocated children, the specific interaction pattern is interpreted cautiously. Taken together, these findings indicate that psychosocial adaptation under poverty-alleviation relocation can be understood as a joint product of external social reception and internal evaluative processes.

### 4.1 The mediating role of subjective SES

The present findings suggest that subjective socioeconomic status is linked to the association between perceived discrimination and psychosocial adaptation among children relocated through poverty-alleviation programs. More specifically, greater perceived discrimination was associated with lower subjective socioeconomic status, which in turn was associated with less favorable psychosocial adaptation. This pattern suggests that the experience of discrimination may become psychologically consequential not only because it is distressing in itself, but also because it may be associated with how children locate themselves within the social hierarchy.

This indirect association is theoretically meaningful in the context of poverty-alleviation relocation. For relocated children, discrimination is unlikely to operate merely as an isolated interpersonal slight (Brown and Lohr, 1987). Rather, it may function as a repeated social signal about who is seen as legitimate, valued, and fully belonging in the new environment. In school and community settings, children do not simply encounter others’ attitudes; they also infer from everyday interactions where they stand relative to others (Brown and Lohr, 1987; Mesch et al., 2008; Shrauger and Schoeneman, 1979). Being ignored, devalued, or subtly marked as different may communicate more than momentary rejection. It may imply lower worth, lower status, and reduced entitlement to recognition (Fan et al., 2012). In this sense, perceived discrimination can be understood as a status-relevant social experience that gradually shapes children’s subjective understanding of their place in the local social order.

At the same time, the present findings should not be interpreted as showing that subjective socioeconomic status operates independently of objective family background. Objective household conditions, parental education, and relocation-related characteristics are likely to be associated with children’s perceived social standing after resettlement (Quon and McGrath, 2014). However, the theoretical value of subjective socioeconomic status in the present study lies not in substituting for objective socioeconomic indicators, but in capturing children’s lived appraisal of their relative position in the new social environment. In resettlement settings, this perceived social position may remain psychologically consequential even when families share broadly similar poverty-relocation backgrounds (Tang et al., 2023). Accordingly, the present findings speak more directly to the possible role of perceived social rank than to the unique contribution of subjective socioeconomic status net of all objective socioeconomic conditions (Tan et al., 2020).

This interpretation is consistent with symbolic interaction perspectives, which emphasize that self-perceptions are formed through reflected appraisals and social feedback (Shrauger and Schoeneman, 1979). Children develop an understanding of who they are partly by interpreting how others respond to them. When relocated children repeatedly encounter cues that they are viewed through the lens of poverty, rural origin, or resettlement identity, those cues may be internalized as judgments about their broader social position. Subjective socioeconomic status is therefore not merely a cognitive estimate of family resources; it also reflects children’s interpretation of their relative social visibility, social worth, and life chances within a stratified environment.

The resettlement context may make this process particularly salient. Poverty-alleviation relocation involves movement from remote and often resource-constrained rural communities into newly established settlements located near towns or urban peripheries (Zhao et al., 2026). Such transitions expose children to new comparison standards. In their place of origin, economic disadvantage may have been more widely shared and therefore less psychologically differentiating (Cheng, 2023b). After relocation, however, disparities in housing conditions, parental occupations, consumption patterns, and access to social resources may become more visible in everyday school life (Zhao et al., 2026). Under these conditions, discrimination may intensify social comparison processes and reinforce the perception that one occupies a lower position than others. What matters here is not only objective deprivation, but the child’s lived awareness of relative standing in a new social environment.

The second part of the indirect association is equally important. Subjective socioeconomic status was positively associated with psychosocial adaptation, suggesting that children’s perceived rank in the social hierarchy is linked to how well they adjust across interpersonal, environmental, and psychological domains. This relationship is theoretically plausible because subjective status can shape both emotional and behavioral orientations toward the social world (Quon and McGrath, 2014). Children who perceive themselves as lower in status may approach social situations with heightened vigilance, reduced confidence, and weaker expectations of acceptance (Quon and McGrath, 2014). They may become less willing to participate, less likely to initiate relationships, and more likely to interpret ambiguity as exclusion (Gao et al., 2017; Haller et al., 2017). Over time, these tendencies may be associated with poorer adaptation by narrowing opportunities for belonging, competence, and engagement (Sette et al., 2023; Tian et al., 2015).

Importantly, the mediating effect observed in the present study was statistically significant but modest in magnitude. The indirect effect accounted for 6.25% of the total effect, indicating that subjective socioeconomic status represents a modest but meaningful pathway linking perceived discrimination to psychosocial adaptation. This modest effect size suggests that perceived social positioning is not the dominant mechanism through which discrimination is linked to adaptation. Rather, within the present framework, subjective socioeconomic status should be understood as one component of a broader multi-path process through which discriminatory experiences become linked to adjustment in the resettlement context.

More specifically, discrimination may influence psychosocial adaptation not only by shaping children’s perceived social rank, but also through several parallel mechanisms. First, discriminatory experiences may heighten emotional responses such as distress, shame, or chronic vigilance, which can directly undermine psychological adjustment (Pascoe and Richman, 2009; Schmitt et al., 2014). Second, they may weaken children’s sense of acceptance, belonging, and legitimacy in the new social environment, thereby making adaptation more difficult even apart from perceived social positioning (Fan et al., 2012; Schmitt et al., 2014). Third, they may disrupt social relational processes, such as trust in others, willingness to participate, and access to supportive peer relationships, all of which are central to successful adjustment after relocation (Fan et al., 2012; Pascoe and Richman, 2009). From this perspective, the present findings contribute to a more differentiated understanding of status-based discrimination in the resettlement context by suggesting that its psychological significance lies not in a single dominant pathway, but in the combined operation of several smaller processes that together shape children’s adaptation.

The present findings should also be interpreted in relation to an important tension in the broader poverty-alleviation relocation literature. On the one hand, existing studies suggest that relocation can improve migrants’ material conditions, social integration, and even subjective social status (Cheng, 2023b). For example, longitudinal evidence indicates that poverty-alleviation relocation may raise resettlees’ subjective social status overall, although this pattern is not indefinitely linear and may vary with post-resettlement duration (Tang et al., 2023). Other studies further suggest that post-relocation adaptation often depends on a gradual progression from economic integration to community integration and then to psychological integration, with community support playing a particularly important role in this process (Feng et al., 2024; Pan et al., 2024; Tang et al., 2022). On the other hand, the present study shows that, even in a policy context intended to improve livelihoods, children’s perceived discrimination remains negatively associated with both subjective socioeconomic status and psychosocial adaptation. Taken together, these findings suggest that material upgrading and formal resettlement do not automatically translate into social recognition or status security for children (Tang et al., 2023). In other words, the benefits of relocation may be real at the structural level, while psychosocial adaptation still depends on whether children experience acceptance, dignity, and legitimate belonging in everyday school and community life (Liu et al., 2014).

Overall, the results highlight the importance of understanding adaptation not only as a response to external living conditions, but also as a response to children’s perceived position within the social world they are entering. For children relocated through poverty-alleviation programs, adapting successfully may depend partly on whether the new environment allows them to feel not merely accommodated, but socially respected and legitimately included. Efforts to reduce discrimination may therefore be important not only because they lessen direct psychological strain, but also because they may protect children from developing a diminished sense of social standing in the resettlement context.

### 4.2 The moderating role of CSE

The present study further found that core self-evaluations moderated the association between perceived discrimination and psychosocial adaptation among children relocated through poverty-alleviation programs. More specifically, the negative association between perceived discrimination and psychosocial adaptation was stronger among children with higher levels of core self-evaluations than among those with lower levels. It is important to note that this finding is not theoretically equivalent to the healthy context paradox literature. That literature concerns contextual-level moderators, such as classrooms characterized by lower aggregate levels of peer victimization, whereas the present study examines an individual-level trait, namely core self-evaluations. The mechanisms are therefore logically distinct. In the present study, the issue is not whether a healthier social context heightens the salience of victimization, but whether more positive trait-level self-appraisals alter how strongly children react to discriminatory experiences.

Core self-evaluations are typically regarded as a positive personal resource, reflecting a person’s fundamental sense of worth, competence, and control (Chang et al., 2012). Children with higher core self-evaluations would ordinarily be expected to show better adjustment because they approach challenges with greater confidence and a more positive view of themselves (Elliott et al., 2012). This expectation was also partly supported in the present study, as core self-evaluations were positively associated with psychosocial adaptation overall. However, the moderation findings suggest that the protective value of core self-evaluations is not unlimited. Its function may depend on the social conditions under which children are attempting to adapt.

One way to interpret this result is through the combined lenses of identity threat and expectancy violation. Children with higher core self-evaluations are more likely to perceive themselves as capable, worthy, and deserving of fair treatment. These positive self-appraisals can be psychologically beneficial when discriminatory experiences are infrequent or relatively mild, because they support confidence, persistence, and emotional stability (Swann et al., 2007). However, when children who hold such positive self-views repeatedly encounter devaluation in the social environment, the discrepancy between self-appraisal and social feedback may become especially salient (Chang et al., 2012). In this situation, discrimination may be experienced not merely as an unpleasant event, but as a stronger violation of expectations of fair treatment, social respect, and recognition (Major and O’Brien, 2005). In that sense, high core self-evaluations may remain beneficial under lower levels of social threat, yet become less protective when discriminatory experiences intensify. This observed interaction pattern can therefore be interpreted as an individual-level moderation process in which positive self-appraisals become less protective when discriminatory experiences strongly contradict children’s expectations of fairness and recognition.

Overall, the moderating findings underscore a central point: the adaptive significance of core self-evaluations depends on context. Among children relocated through poverty-alleviation programs, positive self-evaluations may support adjustment under relatively low levels of discrimination, but their protective role appears to weaken when children face more intense experiences of social devaluation. This pattern reinforces the importance of viewing psychosocial adaptation as the joint product of individual resources and the social environments in which those resources are tested.

### 4.3 Practical implications for poverty-alleviation resettlement contexts

The practical implications of the present study are specific to the policy and social context of poverty-alleviation resettlement rather than to child adjustment in a generic sense. First, the findings suggest that follow-up support in resettlement communities should not focus exclusively on housing conditions, infrastructure, or household income. Existing poverty-alleviation relocation research indicates that long-term adaptation also depends on community support, communicative integration, psychological identity, and sustained post-relocation services. In this regard, improving the spatial environment of resettlement communities, ensuring accessible public spaces, and maintaining follow-up support beyond the initial relocation phase may be important for children’s psychosocial adjustment, especially because gains in subjective social status may not remain stable over time.

Second, the present findings point to the importance of school-based reception processes in resettlement areas. If perceived discrimination is linked to poorer adaptation partly through children’s perceived social standing, then schools should not treat relocated children merely as recipients of welfare support or as administratively marked “special groups.” Instead, schools may need to reduce status labeling in everyday practice, provide teacher training on status-based stigma, monitor exclusion and peer discrimination more systematically, and create mixed-group classroom and extracurricular activities that promote equal participation and social recognition. This implication is consistent with evidence that community and social support help poverty-relocation migrants move from improved material living standards toward a stronger sense of membership and place attachment in the new environment.

Third, the moderation findings suggest that psychosocial interventions should not be limited to generic encouragement or positive affirmation. Although strengthening children’s confidence and self-worth remains valuable, the present results suggest that personal resources may become less protective when the surrounding environment continues to communicate devaluation. Accordingly, interventions for relocated children may be more effective when they combine individual support with relationship and context-oriented measures, such as peer mentoring, after-school clubs that mix relocated and local children, family-school-community coordination, and social work services aimed at rebuilding social networks and everyday recognition.

## 5 Limitations

Several limitations of the present study should be acknowledged. First, all variables were measured using self-report questionnaires completed by the participating children. Although several procedural steps were implemented during data collection to reduce potential bias, self-report data may still be subject to social desirability and perceptual biases. In the present study, common method variance was initially assessed using Harman’s single-factor test. Although the results suggested that common method bias was unlikely to substantially affect the findings, this technique has been criticized as a relatively insensitive diagnostic approach. To further address this concern, confirmatory factor analyses were conducted to compare competing measurement models. The results indicated that the hypothesized multi-factor model provided substantially better model fit than the single-factor model, which reduces concerns that the observed relationships were primarily attributable to common method variance. Nevertheless, future research may benefit from incorporating multiple sources of data, such as teacher reports, parent reports, or observational measures, to strengthen measurement validity.

Second, the cross-sectional design of the study limits the ability to draw causal conclusions regarding the relationships among perceived discrimination, subjective socioeconomic status, core self-evaluations, and psychosocial adaptation. Although the proposed mediation and moderation mechanisms were theoretically grounded, the temporal ordering of these variables cannot be definitively established. Longitudinal or experimental research designs would therefore be valuable for examining how these processes unfold over time and for strengthening causal inference. Relatedly, participants were recruited from six schools using a cluster sampling strategy within poverty-alleviation resettlement areas. Although this sampling approach allowed the study to capture a relatively large sample of relocated children, clustering within schools may introduce potential non-independence in the data. Future studies could employ multilevel analytical approaches or collect data across a broader range of resettlement contexts in order to more fully account for school-level or community-level influences.

Third, another limitation concerns potentially unmeasured background confounds. The present study did not include objective household economic conditions, parental education, time since relocation, or prior residence characteristics. These factors may plausibly be associated with perceived discrimination, subjective socioeconomic status, and psychosocial adaptation. Accordingly, the present analyses should not be interpreted as isolating the unique role of subjective socioeconomic status independent of all objective socioeconomic and relocation-history variables. Future research should incorporate both objective socioeconomic indicators and relocation-related background characteristics in order to examine whether the present pattern remains robust when these factors are explicitly modeled.

Finally, although the present study defined perceived discrimination as status-based discrimination in the resettlement context, the six-item measure used here was designed to capture children’s overall perception of unfair treatment and devaluation rather than to distinguish among specific subtypes of stigma. Accordingly, the present findings should not be interpreted as isolating the unique effects of poverty-based stigma, rural–urban status discrimination, or resettlement identity labeling. In addition, the interpretation of the counterintuitive moderation finding should remain cautious. Because the present data are cross-sectional, the temporal ordering among perceived discrimination, core self-evaluations, and psychosocial adaptation cannot be firmly established, and alternative explanations such as reverse temporal ordering cannot be fully excluded. All focal constructs were assessed using self-report measures, which also leaves open the possibility of shared-method influences. Moreover, the present study did not formally test measurement invariance or differential item functioning of the discrimination and core self-evaluation measures across levels of perceived discrimination. Future research should therefore develop more context-specific multidimensional measures, incorporate multi-informant assessments, and conduct psychometric invariance testing in order to determine whether the present moderation pattern reflects a robust developmental process rather than context-specific measurement features.

Despite these limitations, the present study contributes to the literature by examining the psychological processes linking perceived discrimination and psychosocial adaptation among children relocated through poverty-alleviation initiatives. By identifying both subjective socioeconomic status and core self-evaluations as relevant psychological mechanisms, the study provides insights that may inform future research and intervention efforts aimed at supporting the adaptive development of relocated children.

## Data Availability

The original contributions presented in the study are included in the article/supplementary material, further inquiries can be directed to the corresponding author/s.
